# Unsaturated Fatty Acids Control Biofilm Formation of *Staphylococcus aureus* and Other Gram-Positive Bacteria

**DOI:** 10.3390/antibiotics9110788

**Published:** 2020-11-08

**Authors:** Kamila Tomoko Yuyama, Manfred Rohde, Gabriella Molinari, Marc Stadler, Wolf-Rainer Abraham

**Affiliations:** 1 Chemical Microbiology, Helmholtz Centre for Infection Research (HZI), Inhoffenstraße 7, 38124 Braunschweig, Germany; kamilatomoko@gmail.com; 2 Central Facility for Microscopy, Helmholtz Centre for Infection Research (HZI), Inhoffenstraße 7, 38124 Braunschweig, Germany; Manfred.Rohde@helmholtz-hzi.de (M.R.); Gabriella.Molinari@helmholtz-hzi.de (G.M.); 3 Microbial Drugs, Helmholtz Centre for Infection Research (HZI), Inhoffenstraße 7, 38124 Braunschweig, Germany; Marc.Stadler@helmholtz-hzi.de; 4 Department of Bioinformatics and Biochemistry, Technische Universität Carolo-Wilhelmina zu Braunschweig, BRICS—Braunschweig Integrated Centre of Systems Biology, Rebenring 56, D-38106 Braunschweig, Germany

**Keywords:** fatty acid, *Hypoxylon fragiforme*, biofilm inhibition, Gram-positive bacteria, *Staphylococcus aureus*

## Abstract

Infections involving biofilms are difficult to treat due to increased resistances against antibiotics and the immune system. Hence, there is an urgent demand for novel drugs against biofilm infections. During our search for novel biofilm inhibitors from fungi, we isolated linoleic acid from the ascomycete *Hypoxylon fragiforme* which showed biofilm inhibition of several bacteria at sub-MIC concentrations. Many fatty acids possess antimicrobial activities, but their minimum inhibitory concentrations (MIC) are high and reports on biofilm interferences are scarce. We demonstrated that not only linoleic acid but several unsaturated long-chain fatty acids inhibited biofilms at sub-MIC concentrations. The antibiofilm activity exerted by long-chain fatty acids was mainly against Gram-positive bacteria, especially against *Staphylococcus aureus*. Micrographs of treated *S. aureus* biofilms revealed a reduction in the extracellular polymeric substances, pointing to a possible mode of action of fatty acids on *S. aureus* biofilms. The fatty acids had a strong species specificity. Poly-unsaturated fatty acids had higher activities than saturated ones, but no obvious rule could be found for the optimal length and desaturation for maximal activity. As free fatty acids are non-toxic and ubiquitous in food, they may offer a novel tool, especially in combination with antibiotics, for the control of biofilm infections.

## 1. Introduction

Most microorganisms are organized in aggregates, called biofilms [1]. These biofilms are sessile clusters of microbial cells that are attached on biotic and abiotic surfaces. They consist of a complex matrix containing the cells embedded in extracellular polymeric substances, comprising polysaccharides, proteins and DNA. Biofilms confer protection and resistance against external stress, e.g., desiccation, osmotic stress, radiation, the host immune system or toxic chemicals, e.g., antibiotics [2,3]. Pathogens organized in biofilms are difficult to treat due to their optimal protection. They develop diverse strategies to tolerate high concentrations of antibiotics which, consequently, lead to persistent chronic infections [4,5]. However, the tolerance against antibiotics can be reversed and the cells become again susceptible to antibiotics if the biofilm is dispersed and the cells return to a planktonic lifestyle [6]. The dispersion of established biofilms combined with antibiotic treatments can thus become an alternative approach for the control of biofilm infections [7].

All higher organisms are threatened by biofilm infections and respond through several strategies to counteract them. Fungi are known to produce a multitude of bioactive diverse secondary metabolites, such as antibacterial [8], antifungal [9], antiviral [10], antitumor [11], cholesterol-lowering [12] or antibiofilm compounds [13]. The fungus *Hypoxylon fragiforme* is a member of the Hypoxylaceae (Ascomycota), characterized by nodulisporium-like anamorphs whose secondary metabolite profile changed during the stromatal ontogeny [14,15]. Previously, we reported that in malt extract and potato dextrose media, the species synthesized sclerin and its diacid [16] which could inhibit biofilms of the pathogen *Staphylococcus aureus.* In rice and minimal medium, the metabolite spectrum changed and mainly cytochalasans were produced [17]. We further investigated the diversity of metabolites produced on rice and report here that one of these metabolites, linoleic acid, is effective in the inhibition of *Staphylococcus aureus* biofilms and those of other pathogens, preventing their formation. To shed some light on the structure–activity relationship further, fatty acids were tested, and the results are reported here.

## 2. Results

The fungus *Hypoxylon fragiforme* was collected in the Harz Mountains (Germany) and tested for its ability to inhibit bacterial biofilm formation. After the fermentation of *H. fragiforme* on rice medium, the metabolites were extracted using ethyl acetate and purified using preparative liquid chromatography. Purified compounds were tested against the biofilm formation of a methicillin-sensitive clinical isolate of *Staphylococcus aureus* (MSSA). The minimum inhibitory concentrations (MIC) of the active compounds were determined and only compounds showing biofilm inhibition below their respective MIC concentrations were investigated further. Sclerin, its diacid [16], the cytochalasan L-696,474 and its 21-*O*-deacetyl-derivative were actively inhibiting the formation of *Staphylococcus aureus* biofilms [17] and also another compound. Mass spectra, ^1^H- and ^13^C-NMR and comparison of its retention time in gas chromatography with an authentic standard led to its identification as linoleic acid.

**Figure 1:**
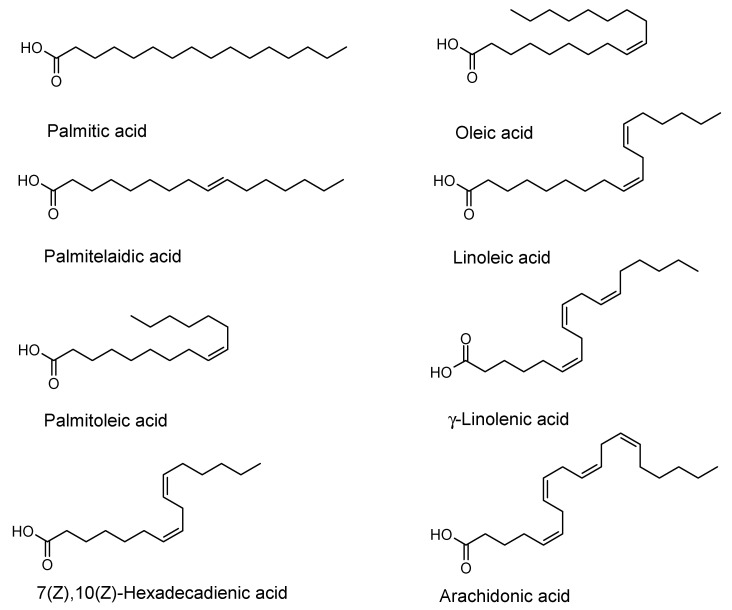
Fatty acids tested range from the C_16_-fatty acids palmitic, palmitelaidic, palmitoleic and 7(Z),10(Z)-hexadecadienic acid, and the C_18_-fatty acids oleic, linoleic and γ-linolenic acid to the C_20_-fatty acid arachidonic acid.

Antibiofilm activities of fatty acids have only been reported for some cis-2-alkenoic acids with 10–14 carbon atoms [18] and some structural rules have been deduced. This has not yet been undertaken for C_18_-fatty acids; therefore, we tested several structurally related fatty acids for their ability to inhibit the biofilm formation of several bacterial pathogens. From the C_16_-fatty acids, saturated palmitic acid, the mono-unsaturated acids palmitelaidic and palmitoleic acid and the double-unsaturated 7(Z),10(Z)-hexadecadienoic acid were chosen for antimicrobial and antibiofilm experiments. Of the homologous C_18_-fatty acids, mono-unsaturated oleic acid, di-unsaturated linoleic acid and tri-unsaturated γ-linolenic acid were selected together with the tetra-unsaturated C_20_-fatty acid arachidonic acid (Figure 1). These fatty acids were purchased from suppliers as indicated in the Materials and Methods section. For the saturated palmitic acid, no biofilm inhibition of any of the strains was observed.

We wanted to discern between antibacterial activity and the inhibition of biofilm formation. To achieve this goal, we determined first the minimum inhibitory concentration (MIC) which is defined as the lowest concentration of a fatty acid which prevented visible growth of a strain. A two-fold dilution of the respective fatty acid was started from 256 µg mL^−1^ against the pathogenic bacteria *Staphylococcus aureus* DSM 1104, *Staphylococcus epidermidis* ATCC 35984, *Bacillus cereus* DSM 626, *Streptococcus mutans* UA59, *Pseudomonas aeruginosa* PA14 and *Escherichia coli* MT102. Where no growth-inhibiting effect at even the highest concentration tested was seen, MIC was set to >256 µg mL^−1^ and not further investigated. All selected fatty acids showed no antimicrobial effects against the Gram-negative bacteria tested, revealing an MIC higher than 256 µg mL^−1^ (Table 1). The same was found for most Gram-positive bacteria and fatty acids. The most susceptible strain was *Bacillus cereus* DSM 626. Here, linoleic acid had a bacteriostatic effect at an MIC of 32 µg mL^−1^, while 7(*Z*),10(*Z*)-hexadecadienoic acid was bactericidal at an MIC as low as 16 µg mL^−1^. 7(*Z*),10(*Z*)-hexadecadienoic acid acted as bactericidal at an MIC of 32 µg mL^−1^ on *Staphylococcus aureus* DSM 1104, while both linoleic acid and γ-linolenic acid had no bactericidal but rather bacteriostatic effects at 64 and 128 µg mL^−1^, respectively. *Staphylococcus epidermidis* was sensitive against 7(*Z*),10(*Z*)-hexadecadienoic acid which acted as bacteriostatic at MIC 16 µg mL^−1^ (Table 1).

The next step was the determination of biofilm inhibition at sub-MIC concentrations, where biofilm inhibition was recorded at different fatty acid concentrations (Table 1). The test results showed that most fatty acids could prevent the biofilm formation of Gram-positive bacteria at sub-MIC concentrations. *S. mutans* was here the most resistant species, where only 7(Z),10(Z)-hexadecadienoic acid could reduce its biofilm formation by 38% at 128 µg mL^−1^ compared to the untreated control. With the exception of palmitic acid and oleic acid, all tested fatty acids could reduce the biofilm formation of *S aureus*, some of them even down to a concentration of 4 µg mL^−1^. The biofilm formation of the pathogen *Staphylococcus epidermidis* was less sensitive against various fatty acids than that of the closely related *S. aureus*. Although *B. cereus* was the most sensitive species for the antimicrobial effect of the fatty acids, some of them could inhibit its biofilms even at sub-MIC concentrations.

**Figure 2:**
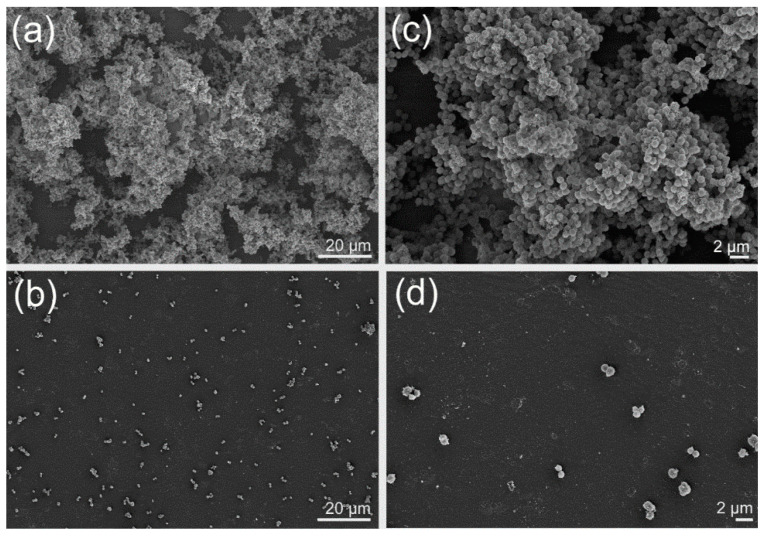
Field emission scanning electron microscopic (FESEM) images at different magnification steps (**b**) and (**d**) of *Staphylococcus aureus* biofilms treated with 64 µg mL^−1^ γ-linolenic acid (64 µg mL^−1^) compared to the negative control (**a**,**c**). Scale bar for (**a**) and (**b**) is 20 µm, for (**c**) and (**d**) it is 2 µm. Please note that in order to get a larger area covered with biofilm, 8-well plates were used here instead of the 96-well plates used for the determination of antibiofilm activities.

Since *S. aureus* was clearly the most susceptible species in the test panel, the effect of fatty acids on its biofilm architecture was studied. When treated with 64 µg mL^−1^ of γ-linolenic acid, both the clustering of cells and the colonization of the substratum were strongly reduced (Figure 2). These effects may point to an interference of the fatty acid with the extracellular polymeric substances (EPS) production of the cells.

The Gram-negative bacteria were found to be more resistant than Gram-positive bacteria. While none of the tested fatty acids inhibited the biofilm formation of *P. aeruginosa,* the biofilm formation of *E. coli* was inhibited by 7(Z),10(Z)-hexadecadienoic acid by 62% and 47% at 128 and 32 µg mL^−1^, respectively. In addition, palmitelaidic acid had a small effect on the same bacterium, inhibiting 25% of biofilm formations at 256 µg mL^−1^. Although almost all fatty acids could inhibit the formation of biofilms, none had any effect against pre-formed biofilms.

## 3. Discussion

Fatty acids are ubiquitous in nature and have shown many biological activities, such as antimicrobial [19], antifungal [20], antiviral [21], nematicidal [22] or anticancer activities [23]. Recently, linoleic acid has also been identified as essential for the sexual cycle of the pathogen *Toxoplasma gondii* in the feline gut [24] or the induction of a memory response of increased pathogenicity of *Pseudomonas aeruginosa* during its passage through the host [25]. These bioactivities are, however, mostly weak and require fatty acid concentrations well above the upper limit of 256 µg mL^−1^ chosen in our study. Our finding that most fatty acids were not antimicrobial at moderate concentrations is in accordance with the literature [26,27]. Herein, we studied the effect of long-chain fatty acids at sub-MIC concentrations on the inhibition of the biofilm formations of pathogenic bacteria.

From the results, it became obvious that biofilms of Gram-positive bacteria are more susceptible to fatty acids than those of Gram-negative bacteria. This concerns not only lower effective compound concentrations but also a broader spectrum of active fatty acids. Many fatty acids showed higher toxicity (lower MIC) to *B. cereus* than to the other strains. To this species, linoleic acid, followed by arachidonic acid, possessed antibiofilm activity at sub-MIC levels. 7(Z),10(Z)-hexadecadienoic acid, followed by linoleic and γ-linolenic acid, was the most bioactive fatty acids for all Gram-positive bacteria (Table 1). Fatty acids at low concentrations had little effect on biofilms of the oral pathogen *S. mutans*. Interestingly, while linoleic acid was quite effective in blocking the biofilm formation of *S. aureus*, it acted only weakly on the closely related *S. epidermidis*. Lee et al. [28] also reported the inhibition of staphylococcal biofilms by linoleic acid, linolenic acid and oleic acid, but in higher concentrations (0.01%) than used here. These differences are less than two-fold and can probably be explained by the differing strains in these two investigations. The higher unsaturated fatty acids cis-4,7,10,13,16,19-docosahexaenoic acid and cis-5,8,11,14,17-eicosapentaenoic acid also showed activity against *S. aureus* biofilms [29].

Several studies focused on the antimicrobial mode of action of fatty acids in *S. aureus*. Parsons et al. demonstrated rapid membrane polarization and the release of solutes and peptides into the medium upon fatty acid treatment. They identified teichoic acids in the bacterial cell wall as being essential for the structure-specific antimicrobial effects of unsaturated fatty acids in *S. aureus* [30]. Increased cell disruption and release of proteases were detected when methicillin-resistant *S. aureus* (MRSA) was grown with 50 µM linoleic acid [31,32]. It has been reported that both oleic and linoleic acid inhibit the fatty acid synthesis of *S. aureus* [33], however, this cannot explain the effect on biofilm formation as oleic acid had no effect but linoleic acid was one of the most active fatty acids in our assays.

The biofilm formation of Gram-negative bacteria proved to be more resistant to fatty acids than that of Gram-positive ones. 7(Z),10(Z)-Hexadecadienoic and palmitelaidic acids showed moderate antibiofilm activity against *E. coli*, inhibiting 46.8% and 25.2% of the biofilm formations, respectively, at sub-MIC concentrations. None of the tested fatty acids had any effect on the inhibition of *P. aeruginosa* PA14 biofilms at moderate concentrations (256 to 4 µg mL^−1^). A much higher concentration of 5 mg mL^−1^ of linoleic acid was necessary for a reduction in the biofilm formation of *Pseudomonas aeruginosa* and for the reported induced memory response during infection of the model host *Galleria mellonella* [25]. Some fatty acids such as linolenic acid combined with tobramycin were reported to interfere at higher concentrations with quorum sensing and inhibited the biofilm formation of *P. aeruginosa* [34]. Soni et al. reported the inhibition of the quorum-sensing autoinducer-2 in their model system *Vibrio harveyi* BB170 by linoleic acid, oleic acid, palmitic acid and stearic acid at concentrations of 250 µm mL^−1^ or higher [35].

No simple picture emerged when comparing the structures of fatty acids with their bioactivities. The poly-unsaturated fatty acids demonstrated higher antibiofilm and antimicrobial activities when compared with the saturated one, palmitic acid, which did not display any activity. Further, a species-specific inhibition of biofilm formation was observed in the limited set of the tested bacteria. While the saturated fatty acid palmitic acid did not have any effect, the simple rule of the more double bonds, the higher the activity did not hold. There seems to be an optimal chain length because the longest fatty acid, arachidonic acid, was less active than the shorter and less unsaturated fatty acid 7(Z),10(Z)-hexadecadienoic acid. While palmitoleic acid could moderately inhibit biofilms of *S. aureus*, the homologous oleic acid had no activity. This corroborates the findings of Parsons et al. [30], who demonstrated that this structure specificity depended on the presence of teichoic acids in the cell wall and that the non-toxic oleic acid became toxic in a strain lacking teichoic acids in the cell wall. It became very evident that the fatty acids were species-specific with the highest activities against Gram-positive bacteria and here *Staphylococcus aureus*.

Further research is needed on whether biofilms of other pathogens could be controlled by fatty acids and whether this reduction in biofilms also causes stronger pathogenicity, as reported for *S. aureus* [32] or *P. aeruginosa* [25]. This, however, could be countered by combining fatty acids with antibiotics.

## 4. Materials and Methods

### 4.1. Reagents

Acetonitrile, chloroform, ethyl acetate and methanol were purchased from J. T. Baker (München, Germany), and D-chloroform, formic acid 98%, Casein-Peptone Soymeal-Peptone (CASO), Potato Dextrose (PD), Luria–Bertani broth (LB), sodium chloride (NaCl), potassium chloride (KCl), potassium dihydrogen phosphate (KH_2_PO_4_), D-methanol and trifluoroacetic acid (TFA) were purchased from Carl Roth GmbH (Karlsruhe, Germany). Bacto malt extract, Bacto peptone and agar were from BD (La Point de Claix, France), and *D*-glucose was from Merck (Darmstadt, Germany). Disodium hydrogen phosphate (Na_2_HPO_4_) was purchased from J. T. Baker (The Netherlands), crystal violet from Fluka (Steinheim, Germany) and tetracycline and potato dextrose agar (PDA) from Sigma Aldrich (Taufkirchen, Germany). The fatty acids hexadecanoic acid (palmitic acid), (9E)-9-hexadecenoic acid (palmitelaidic acid), (9Z)-9-hexadecenoic acid (palmitoleic acid), (9Z)-9-Octadecenoic acid (oleic acid), (9Z,12Z)-octadeca-9,12-dienoic acid (linoleic acid), (6Z,9Z,12Z)-octadeca-6,9,12-trienoic acid (γ-linolenic acid) and (5Z,8Z,11Z,14Z)-Eicosa-5,8,11,14-tetraenoic acid (arachidonoic acid) were purchased from Sigma-Aldrich, St. Louis, MO, USA, while (7Z,10Z)-hexadeca-7,10-dienoic acid came from Larodan Inc., Monroe, LA, USA.

### 4.2. Microorganisms

*Staphylococcus aureus* DSM 1104 (a methicillin-sensitive clinical isolate [36]), *Staphylococcus epidermidis* ATCC 35984, *Bacillus cereus* DSM 626 and *Streptococcus mutans* UA59 were purchased from the German collection of microorganisms and cell cultures (DSMZ). *Pseudomonas aeruginosa* PA14 and *Escherichia coli* MT102 were kindly provided by Prof. Susanne Häußler and Prof. Katharina Riedel, respectively. Bacterial strains were maintained on LB agar at 4 °C.

### 4.3. Isolation, Fungal Identification and Fermentation

The fungus *H. fragiforme* was collected in the Harz mountains (latitude 51°45′21″, longitude 10°32′17″, 724 m) and identified as reported by Yuyama et al. [16]. The fermentation conditions used were the same as described previously [16], where mycelia pellets (5 × 5 mm) from *H. fragiforme* grown on malt extract agar (3% malt extract, 0.5% Bacto peptone and 1.5% agar) were transferred to 2 L Erlenmeyer flasks containing 46 g of rice (Kaufland, Braunschweig, Germany) in 500 mL and static-incubated for 67 days at 22 °C in the dark [16]. After this time, the secondary metabolites were extracted with ethyl acetate and then dried on a rotary evaporator. Then, the compounds were dissolved in acetonitrile for purification and testing for antibiofilm activities.

### 4.4. Minimum Inhibitory Concentration (MIC) and Biofilm Assays

For the determination of MIC and inhibition of pre-formed biofilms, we used the method described by Yuyama et al. [16] and Chepkirui et al. [37], respectively. To establish the MIC of the bioactive compounds, the pre-inocula of bacteria were grown overnight in tubes with Luria–Bertani broth (LB) medium at 37 °C with 180 rpm, adjusted to reach the turbidity of 0.5 McFarland and then transferred to the microtiter plates containing serial dilutions of the bioactive compounds (256 to 2 μg mL^−1^) dissolved in methanol. Methanol and LB medium were used as negative controls and tetracycline (100 μg mL^−1^) as positive control.

Microtiter plates were incubated in the Bioscreen-C automated growth curve analysis system (Oy Growth Curves AB Ltd., Helsinki, Finland). Every 15 min, one OD600 measurement of bacterial growth was recorded for 24 h. Experiments were performed in triplicate. To evaluate bactericidal or bacteriostatic effects, aliquots from different concentrations of the wells, after OD measurements, were inoculated in LB agar for bacterial assays and incubated for 24 h to check the viability of bacteria.

For inhibition of biofilm formations, pre-inocula of bacteria were adjusted to reach the turbidity of 0.5 McFarland standard and 150 µL of the specific medium of each bacterium (CASO with 4% glucose (pH 7.0) for *S. aureus*; LB for *S. epidermidis*, *B. cereus*, *E. coli* and *S. mutans* and CASO for *P. aeruginosa*) was added and transferred to 96-well tissue microtiter plates (TPP, Trasadingen, Switzerland) (only for *S. aureus*)/no-tissue microtiter plates (Falcon Micro Test™, USA) (for the other bacterial strains) in serial dilutions of the bioactive compounds (256 to 3 μg mL^−1^), dissolved in methanol and incubated at 37 °C for 20h [16]. Plates were covered with a sterile adhesive porous paper (Kisker Biotech GmbH, Steinfurt, Germany). After 24 h, the biofilms in the microtiter plates were indirectly measured by staining with crystal violet following a published protocol [38]. To calculate the inhibition of biofilms, the following formula was used: Inhibition of Biofilm formation (%) = ((Negative Control − Blank) − (Sample − Blank))/(Negative Control − Blank) × 100. Methanol was used as negative control. All experiments were performed in triplicates with two repetitions.

Since *S. aureus* was the most susceptible strain, the fatty acids were also tested for their ability to disperse pre-formed biofilms following the protocol of Yuyama et al. [16]. For that, this bacterium was inoculated to reach the turbidity of 0.5 McFarland standard, in 96-well tissue microtiter plates (TPP, Switzerland) containing 150 µL of CASO with 4% glucose with pH 7.0, for 24h at 37 °C. After the incubation, the supernatant was discarded from the wells, washed with phosphate-buffered saline (PBS, 0.8% NaCl, 0.02% KCl, 0.14% Na_2_HPO_4_, 0.02% KH_2_PO_4_) and new medium (150 µL of CASO with 4% glucose) was added together with the serial diluted compounds (256 to 4 μg mL^−1^). Staining, quantification of the biofilm inhibition, replicates and controls were performed as described above.

### 4.5. Purification and Identification of the Compound

Linoleic acid was purified by preparative liquid chromatography (LC) (HPLC 2020, Gilson, Middleton, WI, USA) equipped with a VP Nucleodur 100-7 C18 ec column (125 × 40 mm, 7 µm; Macherey-Nagel, Düren, Germany) using the mobile phase: solvent A: H_2_O (Milli-Q, Millipore, Schwalbach, Germany) with 0.05% trifluoroacetic acid (TFA); solvent B: acetonitrile with 0.05% TFA. The elution gradient started with 95% of solvent B for 3 min, followed by a gradient shift from 95% to 100% of solvent B for 8 min, and finishing with 100% solvent B for 14 min.

The identification of the compounds was confirmed by high-resolution electrospray ionization mass spectrometry (HR-ESIMS), using the same instrumentals setting of Pažoutová et al. [39]. NMR spectra were recorded on a Bruker Ascend 700 spectrometer with a 5 mm TXI cryoprobe (1H 700 MHz, ^13^C 175 MHz) and Bruker AV II-600 (^1^H 600 MHz, ^13^C 150 MHz) spectrometers, such as reported by Yuyama et al. [16]. The fatty acid was identified by comparing the ^1^H and ^13^C chemical shifts to Alexandri et al. [40] and their GC retention time with that of a standard (FAME Mix, Merck, Darmstadt, Germany) [41].

### 4.6. Light and Field Emission Scanning Electron Microscopy

A pre-inoculum of *S. aureus* was grown in CASO with 4% of glucose and adjusted to reach the turbidity of 0.5 McFarland. After that, 150 μL of the bacterial culture was transferred to 8-well Permanox^®^ slides (Thermo Fischer Scientific, New York, NY, USA) and γ-linolenic acid dissolved in methanol was applied into four wells at a final concentration of 64 μg mL^−1^, while methanol was applied in the other four wells and used as a negative control. Plates were covered with a sterile adhesive porous paper (Kisker Biotech GmbH, Steinfurt, Germany) and after 24 h, the effect of the compound on the biofilm formations was evaluated. Biofilms were prepared for field emission scanning electron microscopy as described by Kallscheuer et al. [42].

## 5. Conclusions

There are several reports and a number of patent applications on the antimicrobial activities of fatty acids, but the MICs reported are comparatively high [43]. Here, we demonstrated that several fatty acids could inhibit biofilm formation at sub-MIC concentrations. In fact, linoleic acid was the most active antibiofilm compound produced by the fungus *H. fragiforme*, even more active than its cytochalasans or the recently discovered hybridorubrins [17,44]. The antibiofilm activity exerted by long-chain fatty acids was mainly against Gram-positive bacteria, especially against *Staphylococcus aureus*. The effect seems to be species-, even strain-specific, and no obvious rule could be found as to what would be the optimal length and desaturation of the fatty acids for maximal activity. Here, further studies are needed which should also include antibiotics for the eradication of biofilm infections, as has been reported for some other biofilm-inhibiting compounds [7,45].

## Figures and Tables

**Table 1:** Minimal inhibition concentrations (MIC) and biofilm inhibitions (% compared to the negative control) of the tested fatty acids against several bacteria. No inhibitions at sub-MIC concentrations were found for *Pseudomonas aeruginosa*. Fatty acid concentrations in brackets (µg mL^−1^).

Fatty Acid	*Bacillus cereus*	*Escherichia coli*	*Pseudomonas aeruginosa*	*Staphylococcus aureus*	*Staphylococcus epidermidis*	*Streptococcus mutans*
Palmitic acid	MIC (>256) -	MIC (>256) -	MIC (>256) -	MIC (>256) -	MIC (>256) -	MIC (>256) -
Palmitoleic acid	MIC (>256) -	MIC (>256) -	MIC (>256) -	MIC (>256) 49 ± 2 (128) 54 ± 2 (64)	MIC (>256) -	MIC (>256) -
Palmitelai- dic acid	MIC (>256) -	MIC (>256) 25 ± 4 (256)	MIC (>256) -	MIC (>256) 21 ± 4 (16)	MIC (>256) -	MIC (>256) -
7(Z),10(Z)- Hexadeca- dienoic acid	MIC (16)	MIC (>256) 62 ± 8 (128) 47 ± 21 (32)	MIC (>256) -	MIC (32) 91 ± 2 (32) 70 ± 2 (16) 60 ± 9 (8) 52 ± 10 (4)	MIC (16) 22 ± 14 (8)	MIC (>256) 38 ± 12 (128)
Linoleic acid	MIC (32) 35 ± 15 (16)	MIC (>256) -	MIC (>256) -	MIC (64) 70 ± 9 (64) 71 ± 4 (32) 54 ± 4 (8) 44 ± 2 (4)	MIC (>256) 16 ± 7 (256)	MIC (>256) -
γ-Linolenic acid	MIC (16)	MIC (>256) -	MIC (>256) -	MIC (128) 81 ± 2 (128) 61 ± 6 (64) 58 ± 1 (4)	MIC (>256) 44 ± 19 (128) 27 ± 15 (64)	MIC (>256) -
Arachidonic acid	MIC (>256) 36 ± 0.2 (64)	MIC (>256) -	MIC (>256) -	MIC (>256) 31 ± 4 (256) 35 ± 11 (64)	MIC (>256) -	MIC (>256) -
